# 

**Published:** 1850-03

**Authors:** 


WHOLE SERIES—VOL. VI, NO. VI.

VOL. II.] MARCH, 1850. [No. 6.

Dr. Luther Jewett

### THE NORTH-WESTERN

### MEDICAL AND SURGICAL

### JOURNAL.

PUBLISHED EVERY OTHER MONTH.

AT TWO DOLLARS PER ANNUM IN ADVANCE.

EDITED BY

JOHN EVANS, M.D.,

PROFESSOR OF OBSTETRICS ETC., IN RUSH MEDICAL COLLEGE.

AND

EDWIN G. MEEK, M.D.

CHICAGO AND INDIANAPOLIS:

C. A. SWAN, PRINTER.

1850.

ENLARGED SERIES—VOL. IV, NO. VI.

